# Teen Presentation of Dyssynchronous Left Ventricular Cardiomyopathy Secondary to Wolff-Parkinson-White Syndrome and Right-Sided Septal Pathway

**DOI:** 10.1016/j.jaccas.2025.104400

**Published:** 2025-07-30

**Authors:** Pal V. Shah, Dhruvil A. Patel, Madhusudan Ganigara, Michael G. Earing

**Affiliations:** a Department of Internal Medicine, University of Illinois Chicago College of Medicine, Chicago, Illinois, USA; b Department of Internal Medicine, University of Chicago Medical Center, Chicago, Illinois, USA; c Department of Pediatric Cardiology, University of Chicago Medical Center, Chicago, Illinois, USA; d Department of Cardiovascular Medicine, University of Chicago Medical Center, Chicago, Illinois, USA

**Keywords:** cardiomyopathy, dys-synchrony, septal pathway, Wolff-Parkinson-White syndrome

## Abstract

**Background:**

Wolff-Parkinson-White (WPW) syndrome is associated with an abnormal accessory conduction pathway leading to ventricular pre-excitation and potential re-entrant tachyarrhythmias.

**Case Summary:**

A 13-year-old female presents with shortness of breath, cough, and wheezing for 1 day. She was tachycardic with a left bundle branch block. Echocardiographic findings were notable for dyskinetic septal wall motion with a repeat electrocardiogram uncovering pre-excitation pattern consistent with WPW syndrome and an expected right septal or posteroseptal accessory pathway. Tachycardia had self-resolved, and an outpatient radiofrequency ablation (RFA) was scheduled.

**Discussion:**

WPW syndrome with right septal or posteroseptal accessory pathways causes eccentric septal mechanical activation and may provoke left ventricular (LV) dys-synchrony and dysfunction. Successful RFA is associated with normalization of QRS duration, mechanical resynchronization, and improved LV function.

**Take-Home Message:**

Even in the absence of arrhythmias, our center advocates RFA of right septal or posteroseptal pathways in all patients with significantly decreased LV function.

## Graphical Abstract

**Figure:**
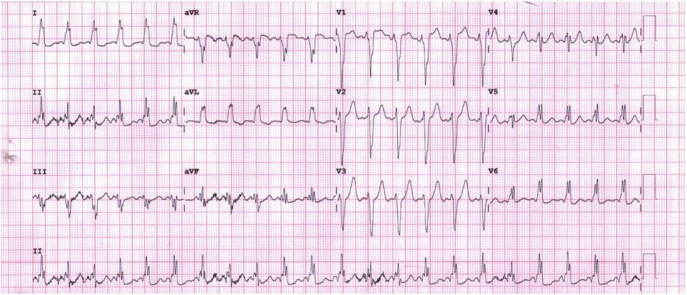

## History of Presentation

A 13-year-old female with no significant past medical history was seen in a community emergency department for shortness of breath, cough, and wheezing for 1 day. She describes being unable to comfortably move around her home without the feeling of needing to catch her breath despite previously never having exercise intolerance before this episode. She appeared to be uncomfortable on the initial examination when taking breaths. She was afebrile but persistently tachycardic with a maximum recorded heart rate of 120. Bilateral lung auscultation revealed wheezing, with no rhonchi or rales. Family history was noncontributory, including no known history of sudden cardiac death or cardiac disease among children in the family.

**Take-Home Messages**

• Wolff-Parkinson-White syndrome can present with nonspecific symptoms that mimic other conditions, such as asthma, and carries the risk of severe arrhythmias and potential left ventricular dysfunction.
• Early diagnosis and treatment with radiofrequency ablation are critical for preventing relapse of complications such as left ventricular dysfunction and improving outcomes.

**Figure 1:**
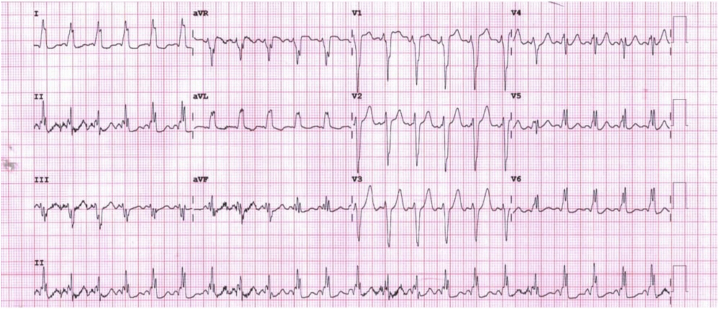
Outside-Hospital ECG: Sinus Rhythm With Left-Axis Deviation, Left Bundle Branch Blockade, and Preventricular Excitation Morphology Preceding Each QRS Complex ECG = electrocardiogram.

A chest radiograph did not reveal any evidence of consolidation or infiltration. Serum electrolytes, renal function, and liver function on the complete metabolic panel were normal. A complete blood count revealed a mild leukocytosis. A respiratory viral screening panel for influenza A, influenza B, respiratory syncytial virus, and SARS-CoV-2 was negative. An electrocardiogram (ECG) obtained because of persistent tachycardia revealed sinus rhythm with a left bundle branch blockade (LBBB) (Figure 1).

Initial therapies including albuterol nebulization, fluid bolus, intravenous magnesium sulfate, and 1 dose of 4 mg of dexamethasone led to some subjective improvement of symptoms. She was also given 1 dose of intravenous ceftriaxone for presumed infection. She was then transferred to our center for further evaluation.

## Past Medical History

This patient and family did not present any known past medical history.

## Differential Diagnosis

In a pediatric patient initially presenting for persistent shortness of breath and diffuse wheezing on examination and no personal medical history, reactive airway disease, including an acute asthma exacerbation, was the leading initial differential diagnosis. Community-acquired pneumonia and viral respiratory tract infection were also considered because of cough and leukocytosis. A persistent sinus tachycardia with the finding of LBBB on ECG also indicated a potential underlying inherent conduction pathway abnormality, including Wolff-Parkinson-White (WPW) syndrome or aberrant conduction pathway alongside inherited sodium channel mutation manifesting as supraventricular tachycardia. Acute inciting cardiac events of arrhythmia can include primary injury to the heart via inflammation—particularly myocarditis or pericarditis. We also considered underlying congenital structural heart pathology and new or inherited genetic arrhythmogenic disorders.

## Investigations

**Figure 2:**
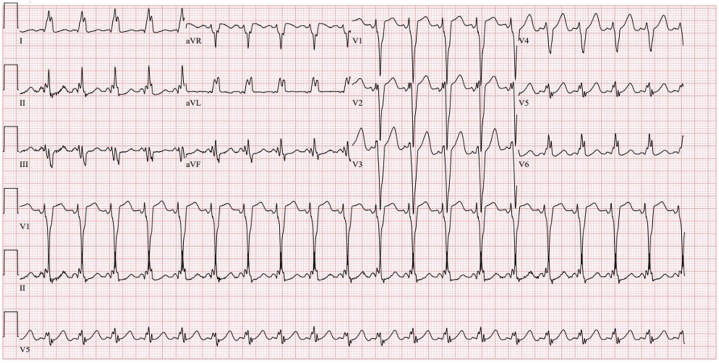
ECG on Initial Presentation Due to Persistent Tachycardia: Sinus Tachycardia With Left-Axis Deviation, Left Bundle Branch Block, and Prolonged QT Interval ECG = electrocardiogram.

Initial ECG performed at the outside hospital before transfer demonstrated tachycardia with preventricular excitation morphology preceding each QRS complex (Figure 1). Upon transfer, repeat ECGs again demonstrated tachycardia with LBBB and PR-interval shortening with intermittent pre-excitation morphology (Figure 2). The N-terminal pro–B-type natriuretic peptide level (211 pg/mL) was elevated with a normal high-sensitivity troponin T level (<6 ng/L). Transthoracic echocardiogram showed relative thinning of the left ventricular (LV) basal and mid-septal wall with dyskinetic motion (Video 1). There were no structural or valvular abnormalities and a borderline ejection fraction of 50% to 55%.

**Figure 3:**
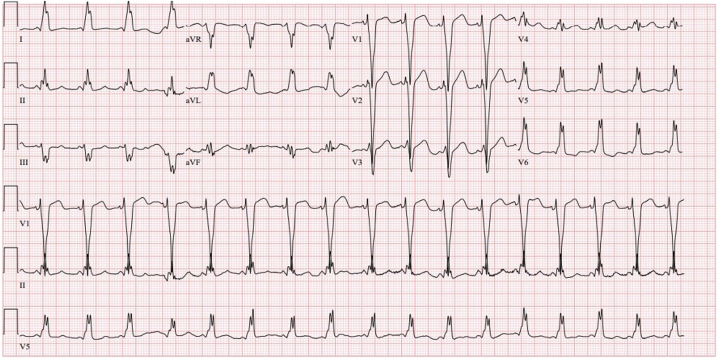
Repeat ECG Due to Persistent Tachycardia: Tachycardia With Left Bundle Branch Block and PR-Interval Shortening With Intermittent Pre-Excitation Morphology ECG = electrocardiogram.

Upon arrival, the patient spontaneously converted to sinus rhythm. The patient had not received an acute rate-control intervention. Another ECG obtained demonstrated WPW syndrome with LBBB (Figure 3).

**Figure 4:**
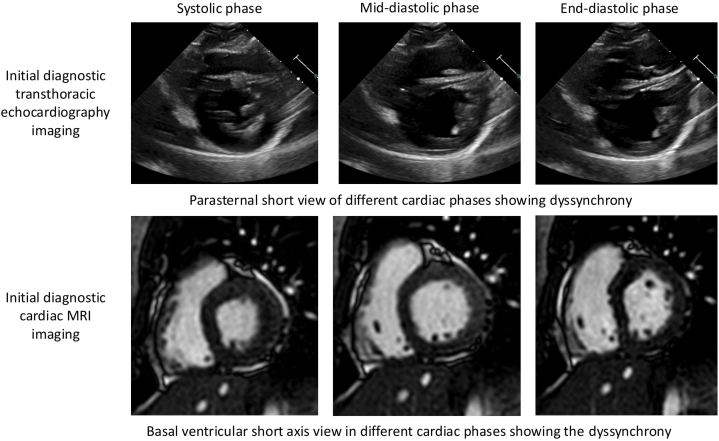
Still Transthoracic Echocardiogram and Cardiac MRI Images Demonstrating Dys-synchrony MRI = magnetic resonance imaging.

Cardiac magnetic resonance imaging performed to assess for other potential ischemic causes of LBBB morphology revealed reduced global LV systolic function with basal and mid-interventricular septal wall dyskinesia consistent with conduction system abnormalities (Videos 2 and 3). There was no evidence of myocardial inflammation, edema, or underlying fibrosing or infiltrative processes (Figure 4).

## Management

Upon arrival, the patient spontaneously converted to sinus rhythm. Resting ECG demonstrated clear evidence of WPW syndrome with a widened QRS complex in an LBBB pattern. Based on the polarity of the delta wave on the resting ECG, the location of the accessory pathway was felt to be a right septal or posteromedial pathway which correlated with both echocardiographic and cardiac magnetic resonance imaging findings with dyskinesia along the mid-interventricular septal wall. Subsequently, the patient underwent elective outpatient radiofrequency ablation (RFA). At the time of the procedure, mapping confirmed the presence of a right posteroseptal accessory pathway that was successfully ablated.

## Outcome and Follow-Up

After ablation, the patient has been asymptomatic without recurrence of tachycardia and evidence of heart failure. At the time of the last follow-up, echocardiogram demonstrated normalization of LV systolic function (Videos 4 and 5).

## Discussion

This case highlights an unusual initial presentation of WPW syndrome in a previously healthy pediatric patient resulting in LV dysfunction. Analysis of the polarity of the delta wave indicated a right septal or posteroseptal accessory pathway. Right septal and posteroseptal accessory pathways result in eccentric septal mechanical activation and may provoke LV dys-synchrony and dysfunction, which fits our patient's clinical presentation.

WPW syndrome is associated with an abnormal accessory conduction pathway leading to baseline ventricular pre-excitation and the potential for life-threatening re-entrant tachyarrhythmias.^1^ The condition is typically identified by the combination of symptomatic arrhythmia with a characteristic pre-excitation ECG pattern of a short PR interval and prolonged QRS complex in sinus rhythm. This distinctive pattern of ventricular pre-excitation is due to the fusion of the accessory pathway and normal electrical condition that runs through the AV node. However, the specific characteristics of the accessory pathway may vary or be transient or concealed.^1^

While a WPW ECG pattern is estimated to be present in as many as 0.25% of the general population, WPW syndrome is far less common, with a prevalence of 0.07% in studies among children aged 5 to 20 years.^2^ In addition, a genetic preponderance has been suggested for WPW syndrome, as 3.4% of those with WPW syndrome have first-degree relatives with a reported pre-excitation syndrome.^3^ Furthermore, there is a known association between WPW syndrome and familial hypertrophic cardiomyopathy correlating with various genetic mutations, including *PRKAG2* and *LAMP2*.^4^ The age of pre-excitation onset correlates with the development of an arrhythmia. Younger patients are more likely to develop arrhythmias, whereas patients who were older than 40 years when pre-excitation was identified were far less likely to develop an arrhythmia.^5^

When symptomatic, WPW syndrome most typically presents as episodic palpitations, chest pain, and presyncope episodes but can include other nonspecific symptoms such as shortness of breath. Presentations involving LV dys-synchrony resulting in subsequent LV dilation and enlargement are an uncommon but potentially life-threatening manifestation of the disease.^6^ The accessory pathway's location is also an important factor for manifesting morphological consequences. In a cohort study by Asakai et al^7^ examining pediatric patients with LV dysfunction and WPW syndrome, 85.7% (12/14) of patients had right-sided accessory pathways distributed across the anteroseptal and both anterior and anterolateral locations, thus demonstrating that right-sided pathways may be a significant predictor for LV dysfunction (OR: 4.32; 95% CI: 1.38-14.18; *P* = 0.012). The location of aberrant right-sided pathways, particularly right septal and posteroseptal accessory pathways, is hypothesized to produce dysfunction through early activation of the LV basal septum, inducing a dys-synchrony with the remaining LV, functionally reducing the LV ejection fraction.

This localized pre-excitation can predispose one to the development of dilated cardiomyopathy often remaining subclinical and sustained across all spectrums of pediatric patients.^8^ WPW syndrome has been shown to be common among children with LV noncompaction and is associated with the occurrence of ventricular dysfunction.^9^ Although the underlying mechanisms of WPW in relation to cardiac remodeling have not been well characterized, these findings further demonstrate that the functional abnormalities can either develop or be exacerbated by WPW in pediatric patients. According to the PACES/HRS Expert Consensus Statement on the Management of the Asymptomatic Young Patient with a Wolff-Parkinson-White (WPW, Ventricular Preexcitation) Electrocardiographic Pattern clinical practice guidelines, RFA treatment for WPW syndrome in pediatric patients has emerged as a widely considered first-line treatment for the resolution of further arrhythmogenic activity.^10^ It also has been proposed to be an appropriate prophylactic strategy to prevent serious arrhythmogenic sequelae irrespective of symptomatology, although long-term follow-up and outcomes remain to be appropriately characterized.

## Conclusions

This case underscores the importance of considering WPW syndrome in pediatric patients with unexplained cardiac dysfunction in the setting of tachycardia or atypical symptoms. Prompt recognition and appropriate management, including referral for RFA, are essential to prevent potentially life-threatening complications and restore normal cardiac function.

## Funding Support and Author Disclosures

The authors have reported that they have no relationships relevant to the contents of this paper to disclose.

## Appendix

Video 1 Transthoracic Echocardiogram: Initial Bedside Echocardiogram Study Showing LV Wall Thinning and Septum Dys-synchrony

Video 2 Cardiac Magnetic Resonance Imaging (MRI): 4-Chamber MRI Study With Visual Demonstration of Pre-excitation Pattern

Video 3 Cardiac Magnetic Resonance Imaging Short-Axis View Illustrating Septal Dyskinesis

Video 4 Transthoracic Echocardiogram: Apical 4-Chamber Image Taken at the Time of the Last Follow-Up After Successful Radiofrequency Ablation Demonstrating Normalization of Left Ventricular Systolic Function

Video 5 Transthoracic Echocardiogram: Short-Axis Image Taken at the Time of the Last Follow-Up After Successful Radiofrequency Ablation Demonstrating Normalization of Left Ventricular Systolic Function
